# Zinc Oxide-Encapsulated Copper Nanowires for Stable Transparent Conductors

**DOI:** 10.3390/nano13192659

**Published:** 2023-09-28

**Authors:** Bo Wang, Shihui Yu, Liang Huang

**Affiliations:** 1 Department of Electrical Engineering and Automation, Luoyang Institute of Science and Technology, Luoyang 471023, China; ysh728@126.com; 2 National Laboratory for Optoelectronics, School of Optical and Electronic Information, Huazhong University of Science and Technology, Wuhan 430074, China; huangliang421@hust.edu.cn

**Keywords:** composite materials, electrical properties, thin films, nanowire, transparent conducting

## Abstract

Cu nanowire (NW)-based transparent conductors are considered to be highly promising constituents of next-generation flexible transparent electronics. However, the fast oxidation of copper under ambient conditions hinders the use of Cu NWs. Herein, we demonstrate a low-cost and scalable approach for preparing a ZnO shell on the surface of Cu NWs under ambient conditions. The covered ZnO shells enhance the oxidative stability of Cu NWs. The optical and electrical properties of ZnO@Cu NWs remain similar to the original performance of the Cu NWs (for example, before encapsulating: 13.5 Ω/sq. at 84.3%, after encapsulating: 19.2 Ω/sq. at 86.7%), which indicates that encapsulation with a ZnO shell enables the preservation of the transparency and conductivity of Cu NW networks. More importantly, the ZnO@Cu NWs exhibit excellent stability in terms of long-term storage, hot/humid environments, and strong oxidizing atmosphere/solution.

## 1. Introduction

Transparent conductors have been of significant technological importance because of their wide range of applications in optical–electrical devices such as transparent heaters, electrochromic windows, solar cells, displays, touch panels, light-emitting diodes (LEDs), electrochromic windows, and more [1,2,3,4,5]. A currently available commercial transparent conductor material is indium tin oxide (ITO), which is used because of its excellent optical and electrical properties [6,7]. Unfortunately, the scarcity and high cost of In lead to high prices in terms of ITO films [7,8]. Particularly, the high-temperature processing required for deposition and inherent brittleness limit the use of ITO in next-generation flexible electronics [9,10]. Therefore, great efforts have so far been made to develop alternative materials and composites [4,11,12,13]. Metal nanowires (NWs), especially Cu NWs, possessing good optical–electrical properties, excellent mechanical flexibility and sufficiently low cost, have been considered highly promising constituents of next-generation flexible transparent electronics [14,15,16]. However, Cu NWs are prone to oxidation because of their high aspect ratio, resulting in rapid deterioration of performance [17,18,19]. This factor limits their practical applications; therefore, enhancing the stability of Cu NWs has become an important research direction at present.

Some research efforts have led to improving the stability of Cu NWs [20,21,22]. Among these attempts, covering the Cu NWs with a dense protective layer is one of the most effective methods. Mehta et al. reported a scalable technique for the low-temperature deposition of graphene around Cu NWs and observed a strong enhancement in terms of the thermal conductivity of graphene-encapsulated Cu NWs compared to uncoated Cu NWs [23]. However, graphene is relatively expensive and cannot be prepared on a large scale. PEDOT:PSS was used by Saeed et al. to improve the stability of Cu NWs [24]. Unfortunately, the hydrophilicity of PEDOT:PSS means that the transparent conductor cannot work in the atmosphere for a long time. Yang et al. prepared Al-doped ZnO/Cu NW-based transparent composite electrodes via RF magnetron sputtering and spin coating techniques [25], which effectively improved the stability of Cu NWs; however, the inherent brittleness of the oxide layer limits the mechanical flexibility of the transparent electrodes. Several methods, mostly by developing a core–shell structure that covers Cu NWs with a metal shell, have been explored to enhance the oxidation resistance of Cu NWs. Au [26], Ni [27], Pt [28], Ag [29] and Ti [30] have been used as corrosion inhibitors. However, previous methods have often been complicated and costly. Therefore, developing a low-cost and simple protective strategy remains a significant challenge.

Recently, our group prepared SnO_2_ [31] and NiO [32] shells on the surface of Cu NWs by using an all-solution process. These shells significantly enhance stability without compromising optical and electrical performances. However, the excessively long growth time of SnO_2_ and NiO shells raises preparation costs. ZnO is a wide-bandgap semiconducting material and possesses excellent thermal stability, chemical stability, and corrosion resistance [33,34]; thus, it often is used as a green inhibitor for corrosion protection of metals [35,36,37]. Herein, we demonstrate a simple deposition method for a ZnO nano-shell on the surface of Cu NWs by using the ZnF_2_ solution. The ZnO@Cu NW-based transparent conductor holds comparable transmittance and conductivity to the bare Cu NWs. More importantly, the transparent conductor shows enhanced stability when subjected to a series of strong oxidizing environments.

## 2. Experimental Section

Cu NWs and the conductive networks on the PET substrates were prepared via the methods used in our previous work [17]. The Cu NWs are obtained via the hydrothermal method. Firstly, 0.8 mmol of copper chloride, 0.8 mmol of ascorbic acid, and 4.8 mmol of Octadecylamine are added to 140 mL of deionized water, followed by ultrasonic mixing for half an hour. Then, the obtained mixed solution was stirred with a magnet for 5 h. Next, the solution was transferred to a reaction kettle, heated at 120 °C for 20 h, and then cooled to room temperature. The solution was then centrifuged in deionized water, n-hexane and isopropyl alcohol, respectively. The obtained Cu NWs were suspended in isopropyl alcohol at a concentration of 2 mg·mL^−1^.

The Cu NWs turbid liquid was spray-coated on the clean PET substrates to form the conductive permeable networks. The density of Cu NW networks can be controlled by changing the length of the spray-coating times. After drying at 60 °C, the Cu NW networks were immersed in 99% glacial acetic acid for 60 s to remove the organics and oxides on the surface. The obtained conductive networks were immersed in 0.07% wt% ZnF_2_ water solution for 1 h under ambient conditions at room temperature. Afterwards, the prepared ZnO@Cu NW networks were washed with alcohol and then dried at 50 °C.

Field emission scanning electron microscopy (FE-SEM, JSM-6700F, Shizuoka, Japan) was used to test the surface topography. Energy-dispersive X-ray spectroscopy (EDS) attached to FEI Talos transmission electron microscopy (TEM) was used to obtain the mapping image of the element. The transmittance was measured byultraviolet-visible spectrophotometer (Jinghua, Shanghai, China). The electrical properties were examined using a four-point probe instrument (Rick Weiyye, Suzhou China) and Keithley Series 2400 System Source Meter instrument. The flexibility was tested using a flexible tester (FlexTest Mini S-P2, Changsha China).

## 3. Results and Discussion

**Figure 1:**
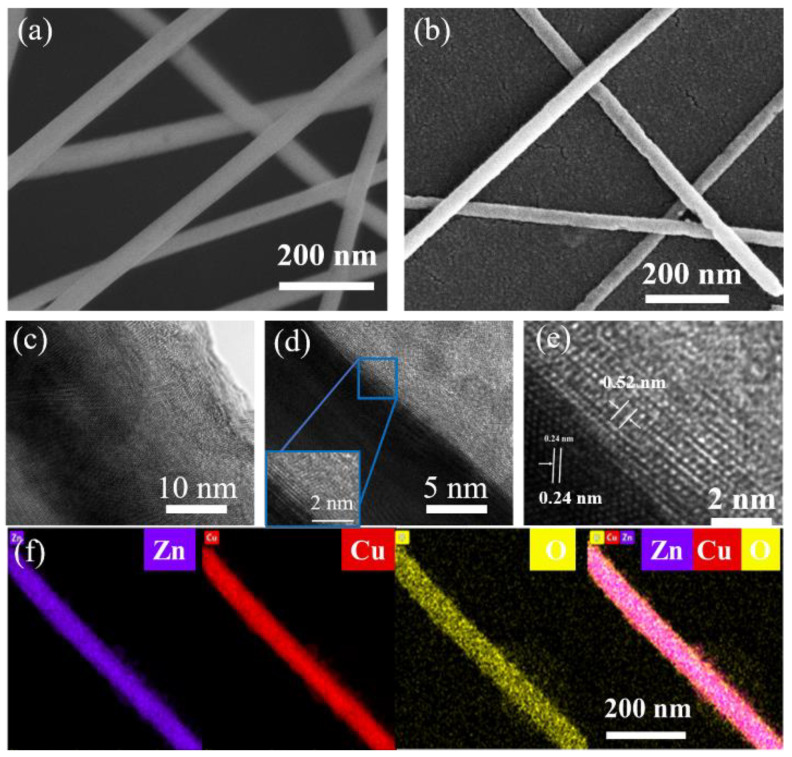
SEM images of (**a**) Cu NW and (**b**) ZnO@Cu NW. HAADF-STEM images of (**c**) Cu NW and (**d**) ZnO@Cu NW. (**e**) Lattice images of core area of ZnO@Cu NW. (**f**) EDS mapping of Zn, Cu, O element distribution.

Figure 1a displays the surface topography of Cu NWs. The surface of the Cu NWs is smooth, and the nanowires are cross-distributed to form a percolating network. Light passes through the gap spaces between the NWs to achieve light transmission; thus, the Cu NW networks are transparent. The Cu NWs are crossed together, and the contacts are formed between the nanowires at the intersection. Free electrons can be transported freely in the NW networks through the contacts between Cu NWs. Therefore, Cu NW networks are conductive. Figure 1b displays the surface topography of ZnO@Cu NWs. The distributional geometry of the ZnO@Cu NW networks is the same as that of Cu NW networks, which suggests that the nanowire networks are not affected by the deposition of ZnO shells. The surface of the ZnO@Cu NWs is smooth, with no obvious differences from the surface of Cu NWs (FE-SEM image in Figure 1a), indicating that the growth of ZnO shells on the surface of Cu NWs is uniform. Figure 1c displays the high-angle annular dark-field image-scanning transmission electron microscopy (HAADF-STEM) image of Cu NW. It can be seen that the Cu NWs have good crystallinity. However, a slight undulating structure can also be observed on the surface of Cu NW. After the fabrication of Cu NWs, the surface of NWs will be slightly oxidized due to the easy oxidation of copper. After the copper oxide is corroded by acetic acid, the surface of the Cu NWs will appear slightly uneven. Figure 1d displays the HAADF-STEM image of ZnO@Cu NW. A new crystal orientation is seen on the surface of ZnO@Cu NWs, which is significantly different from that of the Cu NWs (Figure 1c). The interior structure of ZnO@Cu NW is the same as that of Cu NW; this indicates that the coating reaction of ZnO is only produced on the surface of Cu NWs. As shown in the inset of Figure 1d, there is a layer of ZnO on the surface of Cu NWs that is a few atoms thick, whose thickness is about 2.0 nm. The ZnO shell is evenly distributed around the Cu NWs, which is helpful in improving the stability of Cu NWs. The interplanar spacings of the ZnO shell and Cu core are, respectively, 0.52 nm and 0.24 nm; these are consistent with the lattice spacing of ZnO [38] and Cu [39]. 

Figure 1f shows the EDS mapping images of the ZnO@Cu NW core–shell structure. The Zn and O elements are detected on the surface of Cu NW, confirming the generation of ZnO. Both Zn and O elements are distributed along the Cu NWs, and their distributions are wider than that of Cu; this suggests the conformal, uniform, and complete coating of ZnO on Cu NWs. When a trace amount of ZnF_2_ is dissolved in water, the ZnF_2_ is hydrolyzed to Zn^2+^ and F^−^ ions, resulting in the subacidity of the ZnF_2_ solution. The residual organics and oxides on the surface of Cu NWs are removed by F ions and the surface is cleaned, leading to the production of dangling bonds of Cu. The dangling bonds exist at the surfaces of Cu NWs, which contain lots of free radicals, and therefore, high activity. When the Zn^2+^ and O^2−^ contact the dangling bonds of Cu, a catalytic reaction will take place on the surface of Cu NWs. The adsorbed Zn ions react with the oxygen to form ZnO on the surface of the Cu NWs. The chemical reaction will continue until ZnO covers the surface of the Cu NWs completely. After that, the homogeneous layer of the ZnO shell prevents contact between the Cu and the ZnF_2_ solution, and the dangling bonds is completely consumed. Thus, the formation of ZnO reaction stops. As a result, the thickness of the ZnO shell is controlled at the level of a few atomic layers.

**Figure 2:**
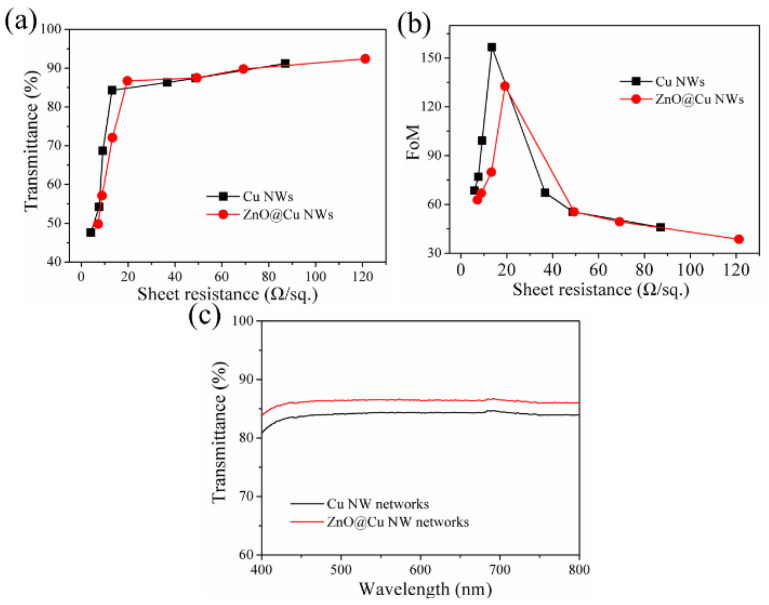
(**a**) Relationship between transmittance (at 550 nm) and sheet resistance for the Cu NWs and ZnO@Cu NW networks. (**b**) Figure of merit value of Cu NW and ZnO@Cu NW networks with different sheet resistances. (**c**) Optical transmittance of Cu NW and ZnO@Cu NW networks in the visible range of 400 to 800 nm.

**Figure 3:**
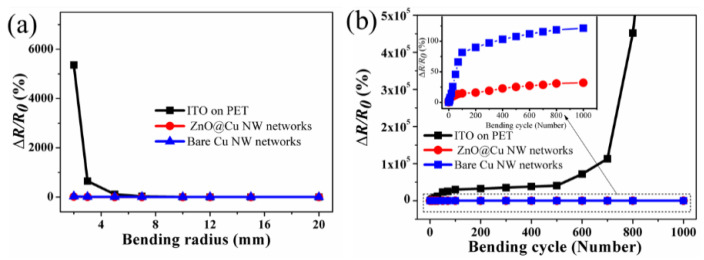
Bending test results of the ZnO@Cu NW networks, bare Cu NW networks and ITO on PET as a function of (**a**) bending radius and (**b**) bending cycle with a bending radius of 5 mm.

Figure 2a shows the relationship between transmittance (at 550 nm) and sheet resistance for the Cu NW and ZnO@Cu NW networks on PET substrates. For the Cu NW networks, the optical transmittance reduces gradually with decreasing sheet resistance; this can be attributed to the augmentation of the network density. When the sheet resistance is 82.7 Ω/sq., the optical transmittance is 91.6%. As the sheet resistance reduces to 15.1 Ω/sq., the transmittance decreases slightly to 85.7%. With the sheet resistance further reduced to 4.1 Ω/sq., the transmittance drops dramatically to 46. 8%. The resistance of the Cu NW networks depends on the line resistance of the Cu NWs and the contact resistance between the Cu NWs. In brief, the resistance of Cu NW networks can be considered as a series of line resistance and contact resistance. Because the line resistance is much lower than contact resistance [3,5], we can regard that the conductivity of Cu NW networks as mainly affected by the contact resistance between the Cu NWs. When the density of Cu NWs is relatively sparse, the cross junctions are few, leading to high sheet resistance. With the increased density of Cu NWs, the cross junctions rapidly increase, reducing sheet resistance. At the same time, because the density of Cu NWs is not high enough, there is no significant decrease in transmittance. When further increasing the density of Cu NWs, the optical scattering and reflection of Cu NWs increase sharply, leading to a drop in transmittance. For ZnO@Cu NW networks, the variation trend of sheet resistance is the same as that of Cu NW networks. When the sheet resistance is 121.3 Ω/sq., the optical transmittance is 91.8%. As the sheet resistance reduces to 8.5 Ω/sq., the transmittance decreases slightly to 50.2%. It is important to note that the sheet resistance increases slightly after coating Cu NW networks with ZnO shells. The Cu NWs are adsorbed on the PET substrate via van der Waals forces, and the adhesion is very poor. When the Cu NW networks are immersed in ZnF_2_ solution for the preparation of ZnO shells, a small amount of Cu NWs fall off from the substrate due to the impact effect of the liquid, leading to a lower density of Cu NWs; thus, the sheet resistance increases, and the optical transmittance rises (as shown in Figure 3).

The work functions of ZnO and Cu NWs are 5.3 eV [40] and 4.6 eV [41], respectively. The ohmic contact will form after ZnO shells contact with Cu NWs due to the N-type conductivity and larger work function of ZnO shells. When the ZnO shells come into contact with Cu NWs, a lot of free electrons flow from Cu NWs to ZnO shells due to the existence of potential barrier height between Cu NWs and ZnO shells. A negative space charge region is formed on the near interface in the ZnO shells, resulting in the generation of a built-in electric field from the ZnO shells to the Cu NWs. In the meantime, the conduction and valence bands of ZnO shells curve downward and curve upward, which makes the surface of the ZnO shells more n-type. After establishing thermodynamic equilibrium, a Fermi-level constant is seen on the interface between ZnO shells and Cu NWs. At this moment, the potential barrier height is reduced to zero, and electrons are injected into ZnO shells from Cu NWs, which leads to the good electrical conductivity of ZnO shells. In addition, due to the very thin thickness of the ZnO shells, dielectric or quantum tunneling will occur, and electrons in the Cu NWs can move through the ZnO shells, enabling the conductivity of the ZnO shells. 

For transparent electrodes, light transmission and electrical conductivity are equally important. It needs a high transmittance and low sheet resistance for the ideal transparent electrode. However, transmittance and sheet resistance show a positive correlation relationship. Therefore, it is very important to balance the optical and electrical properties to assess the quality of the transparent electrode. The figure of merit for the optical and electrical properties is expressed as follows [42,43]:

(1) $$\varphi_{T}=\frac{377}{2\;R_{sh}\left(\frac{1}{\sqrt{T}}-1\right)}$$

where *φ_T_* denotes the figure of merit, *R_sh_* denotes resistance, and *T* denotes the transmittance at 550 nm. The obtained *φ_T_* values are shown in Figure 2b. For the Cu NW and ZnO@Cu NW networks, *φ_T_* values first increase and then decrease with the increase in sheet resistance. For the case of there being a low density of nanowires, the sheet resistance is high due to the lack of connections between NWs, resulting in low *φ_T_* values. With an increased density of nanowires, the nanowires interconnect with each other, creating conductive networks and leading to improved conductivity and increased *φ_T_* values. However, the exorbitant density of nanowire networks will lead to the fact the loss of transmittance is relatively larger than the improvement of the conductivity, causing the drop of *φ_T_* values. The highest *φ_T_* value of ZnO@Cu NW networks is obtained as 132.8, which is close to the highest *φ_T_* value of Cu NWs (156.3). The comparison of *φ_T_* values between the Cu NWs and ZnO@Cu NWs indicates the encapsulation of ZnO shells has almost little impact on the electrical-optical properties of Cu NW networks. 

The conventional ITO is susceptible to cracking when bent because of the intrinsic ceramic brittleness [44,45]. In contrast, Cu NWs show very good bending performance due to the intrinsic flexibility of metal Cu. In order to better replace ITO and make up for the defects of ITO, ZnO@Cu NWs should possess better mechanical flexibility than the ITO. The flexibility of transparent conductors can be evaluated by measuring the resistance variation after the bending tests. Figure 3 displays the bending test results of the ZnO@Cu NW networks, bare Cu NW networks and ITO on PET. The resistance change value can be considered Δ*R/R*_0_ = (*R* − *R*_0_)/*R*_0_; here, *R*_0_ and *R* represent the initial resistance and the resistance after the bending cycle, respectively. Figure 3a exhibits the variation of sheet resistance for ZnO@Cu NWs, bare Cu NW networks and ITO on PET as a function of curvature radius. The resistances of the ZnO@Cu NW and bare Cu NW networks do not change until a curvature radius of 2.0 mm, showing outstanding mechanical flexibility. However, the resistance of ITO increases gradually with a reduced curvature radius and rises significantly to 5360% when a bending radius of 2.0 mm is reached, which is concordant with the reports of ITO cracking after bending tests. Figure 3b exhibits the bending test results of ZnO@Cu NW, bare Cu NW networks and ITO on PET as a function of the bending cycle. The test is carried out 1000 times at a frequency of 1 Hz and with a curvature radius of 5 mm. With the increase in the bending cycle, the resistance of ITO increases. After 1000 bending cycles, ITO thin films lose conductivity due to their brittleness. In contrast, the resistances of ZnO@Cu NW and bare Cu NW networks nearly stay the same. The resistance of bare Cu NW networks is increased by 121%, while the resistance change of the ZnO@Cu NW networks is below 32% after 1000 bending cycles; this can be attributed to the variation in geometrical slipping and delamination at the Cu NW junctions [46,47]. The lower resistance change of ZnO@Cu NW networks compared to that of bare Cu NW networks is because of the reinforcement effect of ZnO shells. 

**Figure 4:**
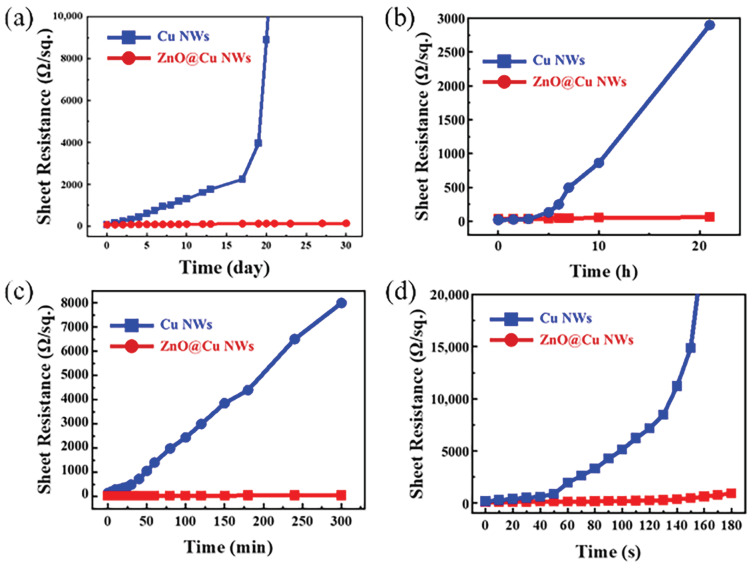
Sheet resistances of Cu NW and ZnO@Cu NW networks with different times (**a**) stored in ambient atmosphere, (**b**) stored in high temperature (85 °C) and high humidity (85%) environment, (**c**) under ultraviolet ozone irradiation, (**d**) immersed in 3 wt% H_2_O_2_ solution.

Stability is a key criterion for transparent electrodes within practical applications. In a natural environment, copper is prone to oxidation, and as an improved scheme of performance of Cu NWs, ZnO@Cu NWs should have better antioxidant properties than bare Cu NWs. Figure 4a shows the sheet resistances at different storage times under ambient atmosphere for the Cu NW and ZnO@Cu NW networks. The sheet resistance of Cu NW networks initially rises slowly within 17 days, then increases sharply from 17 days to 20 days, and beyond the scope of measurement systems after 20 days. The initial increase can be explained by the fact that the surface of Cu NWs is gradually oxidized into CuO or Cu_2_O [48,49]; the Cu NWs become thinner, thus leading to a slow increase in sheet resistance. With the extension of storage time, Cu NWs break and become discrete, resulting in a loss of conductivity caused by the loss of conductive channels. However, the sheet resistance of ZnO@Cu NWs is independent of storage time. The Cu NW and ZnO@Cu NW networks are stored in a high temperature (85 °C) and high relative humidity (85%) environment for 22 h. The sheet resistances of Cu NW and ZnO@Cu NW networks at different storage times are shown in Figure 4b. The sheet resistance of Cu NWs is raised quickly and out of the instrument test range after 22 h owing to the oxidation of copper. In contrast, the ZnO@Cu NW networks maintain almost the initial sheet resistance within the entire test period (24 h), suggesting excellent oxidation resistance. An ultraviolet ozone (UVO) irradiation test was also performed to assess the antioxygenic property of ZnO@Cu NWs. Figure 4c displays the sheet resistances at different irradiation times under UVO irradiation for the Cu NW and ZnO@Cu NW networks. The sheet resistance of Cu NWs rises gradually with the increase in the irradiation time, rising 800-fold after 600 min. However, the sheet resistance of ZnO@Cu NWs changes little throughout the testing period, suggesting that the ZnO shells have a good isolation effect on ozone. Figure 4d shows the sheet resistances of the Cu NW and ZnO@Cu NW networks as a function of immersion time in 3 wt% H_2_O_2_ solution. The Cu NW networks lost conductivity after 160 s. In contrast, the sheet resistance of the ZnO@Cu NWs only increased by 297% after 180 s. Significantly, the sheet resistance of the ZnO@Cu NWs remains basically unchanged after an immersion time of 120 s, and the sheet resistance gradually increases after 120 s. The ZnO shells covering the Cu NW networks prevent contact between ZnO and –O–O–; the peroxide ions cannot pass through the ZnO, thus there is initially no increase in sheet resistance. Longer exposures to H_2_O_2_ solution lead to a slight increase in sheet resistance, which is due to the oxidation of Cu NWs below the ZnO shells caused by that fact that some peroxide ions pass through the ZnO shells. These results confirm that the ZnO shell completely encapsulates the Cu NWs and is sufficiently dense enough to prevent contact of oxygen and water molecules with Cu NWs. Otherwise, oxidation is inevitable. 

**Figure 5:**
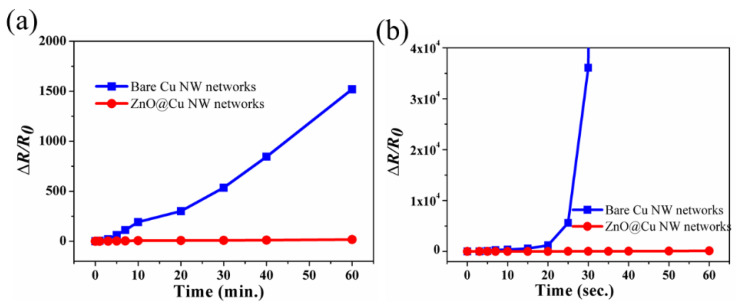
(**a**) Variation in resistance of ZnO@Cu NW networks andbare Cu NW networks after immersion in 1 wt% NaCl solution. (**b**) Variation in resistance of ZnO@Cu NW networks, bare Cu NW networks after immersion in 0.2 wt% Na_2_S solution.

In actual application, transparent electrodes easily come into contact with environments containing sulfides or chloride. Therefore, a transparent electrode should have excellent corrosion resistance. To verify the corrosion resistance, the ZnO@Cu NW networks are immersed in a 1 wt% NaCl solution for a certain time; the variation of the sheet resistance of ZnO@Cu NW networks with immersion time is shown in Figure 5a. After 1 h, the sheet resistance of ZnO@Cu NW networks has changed very little, while the sheet resistance of bare Cu NW networks is increased by 15.2 folds. The results show that the dense ZnO shells can effectively isolate the Cl ions and oxygen from contact with Cu atoms. In addition, metal Cu is extremely susceptible to sulfide in sulfur-containing gases and liquids that produce copper sulfide, which will not only blacken but also affect the electrical conductivity of the Cu NWs [50,51,52]. Therefore, improving the sulfur resistance of Cu NWs is also an important research direction. The ZnO@Cu NW networks and bare Cu NW networks are immersed in a 0.2 wt% Na_2_S solution to measure the change in sheet resistance. As shown in Figure 5b, the sheet resistance of ZnO@Cu NW networks increases only 117% after 1 min. In comparison, the sheet resistance of bare Cu NWs increases 360 times after 30 s and exceeds the scope of the instrument test after 1 min. The ZnO shells protect the Cu from sulfuring molecules on the surface, resulting in the improved stability of ZnO@Cu NW networks. The presence of ZnO shells improves the stability of the bare Cu NWs in saltwater and NaS solutions. However, due to the limitation of zinc oxide’s own instability, more stability tests need to be performed in future work. New and more stable oxide shell materials also need to be further investigated in further work.

## 4. Conclusions

In summary, in order to obtain stable Cu NW-based transparent conductors, ultrathin ZnO@Cu core–shell NWs have been developed through a reliable, low-cost, and scalable solution approach. The ZnO shells are formed uniformly on the surface of Cu NWs through the use of a solution process, which enhances the oxidation resistance of Cu NWs. Unlike bare Cu NWs that are easily oxidized to CuO or Cu_2_O, ZnO@ Cu NWs can survive in various strong oxidizing environments, displaying outstanding antioxidation properties. Moreover, the optical and electrical properties of ZnO@Cu NWs remain similar to that of the original Cu NWs (before encapsulating: 13.5 Ω/sq. at 84.3%, after encapsulating: 19.2 Ω/sq. at 86.7%), which indicates that the encapsulation with a ZnO shell has no effect on the transparency and conductivity of Cu NW networks. In addition, ZnO@Cu NWs also present excellent stability in environments containing sulfides or chloride. We believe this strategy and preparation technology to be broadly applicable to various Cu surfaces.

## Data Availability

Data are available from the corresponding authors upon request.
